# The Short-Term Effects of Transcranial Direct Current Stimulation on Electroencephalography in Children with Autism: A Randomized Crossover Controlled Trial

**DOI:** 10.1155/2015/928631

**Published:** 2015-03-12

**Authors:** Anuwat Amatachaya, Mark P. Jensen, Niramol Patjanasoontorn, Narong Auvichayapat, Chanyut Suphakunpinyo, Suparerk Janjarasjitt, Niran Ngernyam, Benchaporn Aree-uea, Paradee Auvichayapat

**Affiliations:** ^1^Department of Physiology, Faculty of Medicine, Khon Kaen University, Khon Kaen 40002, Thailand; ^2^Department of Rehabilitation Medicine, University of Washington, 1959 NE Pacific Street, P.O. Box 356490, Seattle, WA 98195-6490, USA; ^3^Department of Psychiatry, Faculty of Medicine, Khon Kaen University, Khon Kaen 40002, Thailand; ^4^Department of Pediatrics, Faculty of Medicine, Khon Kaen University, Khon Kaen 40002, Thailand; ^5^Department of Electrical and Electronic Engineering, Faculty of Engineering, Ubon Ratchathani University, Ubon Ratchathani 34190, Thailand

## Abstract

Abnormal synaptic maturation and connectivity are possible etiologies of autism. Previous studies showed significantly less alpha activity in autism than normal children. Therefore, we studied the effects of anodal tDCS on peak alpha frequency (PAF) related to autism treatment evaluation checklist (ATEC). Twenty male children with autism were randomly assigned in a crossover design to receive a single session of both active and sham tDCS stimulation (11 mA) over F3 (left dorsolateral prefrontal cortex). Pre- to postsession changes in a measure of cortical activity impacted by tDCS (PAF) and ATEC were compared between groups. We also examined the associations between pre- and postsession changes in the PAF and ATEC. The results show significant pre- to postsession improvements in two domains of ATEC (social and health/behavior domains) following active tDCS, relative to sham treatment. PAF also significantly increased at the stimulation site, and an increase in PAF was significantly associated with improvements in the two domains of ATEC impacted by tDCS. The findings suggest that a single session of anodal tDCS over the F3 may have clinical benefits in children with autism and that those benefits may be related to an increase in PAF.

## 1. Introduction

Autism is a neurodevelopmental disorder [1] that has a prevalence rate of 62/10000 in the general population [2]. Autism spectrum disorder (ASD) is characterized by three core symptoms: (1) impaired language and communication, (2) deficits in social interactions, and (3) restricted or repetitive behaviors and interests. It is also commonly associated with a number of additional health problems such as sensory abnormalities, sleep disturbances, and gastrointestinal symptoms [3].

The causes and pathophysiology of ASD are not yet clear [4]. Brain imaging studies have found that the volume of right brain structures related to language and social function is greater in the right hemisphere relative to patients' own left hemispheres and relative to normal subjects [5]. Moreover, hypoactivation in the left hemisphere, relative to the right hemisphere, has been found in individuals with ASD [6–9]. In addition, abnormal synaptic maturation, brain connectivity, and mirror neuron dysfunction have all also been purposed as neurophysiological factors that may underlie the symptoms associated with ASD [10–13].

Electroencephalography (EEG) can provide a number of measures of brain activity, including the assessment of functional connectivity between different areas of the brain as well as the magnitude (“power”) of specific oscillations bandwidths, which reflect overall brain activity and different brain states and processes [14]. Such measures could potentially help to further understand the potentially impaired interactions between brain regions in individuals with ASD that have been identified by functional MRI studies [15–21]. In addition, as noted by Wang and colleagues [22], EEG has some specific advantages over other imaging procedures such as MRI. Specifically, EEG can be used with subjects who are younger and with individuals with lower developmental abilities. EEG also has higher temporal resolution than MRI, allows for more movement during the assessment procedures, and is generally more available. Finally, resting-state assessments do not require a specific stimulus or require a specific response or change in activity to be useful. All of these strengths make EEG very useful for assessing brain activity in individuals who may be more severely impaired or too young to be able to comply with the behaviors required for MRI assessment.

In support of the idea that EEGs may be useful for assessing neurophysiological correlates of ASD, Cantor and colleagues found that 11 children with autism evidenced significantly more very slow wave activity, less alpha, and less inter- and intrahemispheric asymmetry, relative to children without ASD, relative to three groups of children without autism, that is, (1) 88 normal children; (2) a matched group of 18 mentally handicapped children; and (3) a group of 13 mentally age-matched normal toddlers [23]. This result was replicated by Chan et al. who found that the average quantitative EEG profile from a sample of 66 children with ASD showed significantly less relative alpha activity than the average profile from a sample of 90 children who did not have ASD [24]. In addition, Murias and colleagues [25] found less coherence between the frontal cortex and other brain areas in the alpha bandwidth in a sample of 18 adults with ASD, relative to a sample of 18 adults without ASD.

Transcranial direct current stimulation (tDCS) provides a weak current, usually ranging from 1 to 2 mA to the scalp using large electrodes, usually for about 20 minutes at a time [26, 27]. Positive current is provided via an anode and negative current via a cathode. The anode lowers the firing threshold of the neurons that lie in the cortex (i.e., increase the activity of those neurons) and the cathode increases the firing threshold of neurons (i.e., inhibits the activity of those neurons) [26, 28]. The effects of tDCS have been shown to last beyond the individual session, with measurable cortical activity facilitation (anode) and inhibition (cathode) lasting for up to 48 hours after stimulation [29].

The frontal lobe is known to play an important role in cognitive, social, and emotional functioning [30]. Moreover, the dorsolateral prefrontal cortex (DLPFC) also plays an important role in working memory [31]. Previous research using proton magnetic resonance spectroscopy (H-MRS) has shown lower levels of N-acetyl aspartate (NAA, a marker of mitochondrial function and neuronal density) in the left DLPFC (F3) of autistic patients, relative to otherwise healthy individuals. The findings suggest that dysfunction in the left DLPFC may be a component of the pathogenesis of autism [32]. This may explain why anodal tDCS stimulation at F3 may improve the effectiveness of autism treatment via its beneficial effects on cognitive processes associated with DLPFC activity, such as attention and memory.

Consistent with these ideas, evidence indicates that anodal tDCS had effects on cortical activity in individual with autism; Schneider and Hopp explored the effects of anodal tDCS applied over the left dorsolateral prefrontal cortex (F3 in the 10/20 system) for 30 minutes in 10 individuals with ASD. They found significant pre- to poststimulation improvements in language acquisition immediately after the tDCS session (effect size [Cohen's *d*] = 2.78, *P* < 0.0005) [33]. In a subsequent study, we sought to replicate and extend these promising preliminary findings by examining the effects of 1 mA of anodal tDCS versus sham tDCS applied over F3 for 20 minutes on 5 consecutive days in a randomized double-blind crossover trial in 20 children with ASD. We assessed autism severity using the childhood autism rating scale (CARS) and autism symptoms using the autism treatment evaluation checklist (ATEC). We found significant improvements from baseline to 7 days after stimulation in both measures [34].

Based on the EEG findings of reduced power in middle-range (alpha) frequencies in individuals with autism [23–25, 35], we reasoned that one possible mechanism of tDCS is that it results in an increase in alpha frequency in the area under the anode, which may reflect an increase in synaptic connectivity, and that this tDCS-related increase in alpha frequency would be associated with symptom improvement in those who receive tDCS. The current study was designed to test these ideas. Specifically, we hypothesized that there would be (1) a greater increase in peak alpha frequency (PAF) pre- to post-tDCS stimulation among a group of children with ASD who receive anodal tDCS over the F3 cortex, relative to a group who receive sham tDCS, and (2) a negative association between change in PAF and change in autism symptoms (specifically, assessing speech/language/communication problems, social subscale problems, sensory and cognitive awareness problems, and health/physical/behavior problems, as measured by the autism treatment evaluation checklist (ATEC) [36], and (3) that the hypothesized increases in alpha frequency and associations between changes in alpha frequency and autism symptoms would be specific to the stimulation site (F3) and would not be found at other electrode sites.

## 2. Materials and Methods

### 2.1. Participant Recruitment and Informed Consent

The participants in the current study were recruited via advertisements at three clinics in Thailand: (1) outpatient Child Neurology Clinic, Child Development Clinic, Khon Kaen University; (2) Child Psychiatric Clinic of the Srinagarind Hospital, Faculty of Medicine, Khon Kaen University; and (3) the Khon Kaen Special Education Center. Clinic physicians described the study procedures to any eligible participants and their parents who expressed an interest in study participation. A psychiatrist confirmed an ASD diagnosis and also ruled out mental retardation based on the DSM-IV TR diagnostic criteria [37].

Study inclusion criteria were (1) male participants with autism; (2) age between 5 and 8 years; and (3) mild to moderate autistic symptoms (CARS score 30–36.5). Study exclusion criteria included (1) mental retardation; (2) use of a pacemaker or other metal device in the body; (3) severe neurological disorders such as a brain tumor or intracranial infection; (4) uncooperative parents or caregivers; (5) being diagnosed with epilepsy; (6) skull defect; and (7) use of other complementary or alternative therapies, such as melatonin, omega 3 fatty acid, casein, and/or a gluten-free diet. Ethical approval based on the Declaration of Helsinki was provided by the Ethics Committee of Khon Kaen University (Identifier number: HE 541409). Before participation, all caregivers provided informed consent.

### 2.2. Study Design

The study was a randomized double-blind controlled placebo (sham tDCS) crossover trial performed for 3 weeks consisting of (1) baseline assessment; (2) a single session of 1 mA anodal or sham tDCS stimulation (depending on order of assignment) for 20 minutes; (3) 1 week of assessment and washout period; (4) another session of 1 mA anodal or sham tDCS stimulation; and (5) a final week outcome assessment. Thus, the study involved 3 weeks of participation. Participants were asked to continue their routine medication and behavioral treatment regimens throughout the duration of the 3-week study.

### 2.3. Randomization and Blinding

Just before the treatment phase, study participants were randomized in a 1 : 1 ratio in blocks of four randomizations (by NN) to receive either (1) active tDCS stimulation or (2) sham tDCS stimulation. Participants were asked to continue their routine medication regimen throughout the trial. The staff who generated the random allocation sequence, enrolled participants, and assigned participants to interventions were not involved in any assessments. After assignment to the intervention groups, the psychologist who carried out the autism assessments (NP) was blind to treatment condition. Because the study participants were also blind to treatment condition, this is a double-blind study.

### 2.4. Active and Sham Transcranial Direct Current Stimulation

We applied tDCS stimulation using a pair of 35 cm^2^ sponge electrodes soaked with 0.9% NaCl solution. A battery-powered constant current stimulator (Soterixmedical, Model 1224-B, New York, USA) had a maximum output of 10 mA. The anode was placed over F3 (international 10-20 EEG electrode placement system) with a goal of targeting the left dorsolateral prefrontal cortex (DLPFC). The cathode (reference) electrode was placed on the shoulder contralateral to the anode. During the 20 minutes of stimulation of each session, the current was gradually increased until we achieved the necessary current (1 mA) and was then decreased at the end of the session.

The tDCS device we used also allowed for masked (sham) stimulation. Specifically, the control switch was covered by an opaque adhesive during stimulation, so that it was not visible to the participants. Moreover, the power indicator which was visible to the participants was lit up during both the active and sham stimulations. However, in the sham stimulation condition, the current was discontinued after 30 seconds [29].

## 3. Measures

Five outcomes were assessed in this study: peak alpha frequency (PAF) and four domains of autism problems as assessed by the ATEC. In addition, any adverse events associated with active and sham stimulation procedures were monitored and recorded.

### 3.1. EEG Recording

EEG data were recorded by trained staff. EEG was acquired from all participants using 32 channels, referenced to Cz, international 10-20 system of electrode placement (Neuvo, Compumedics, Australia with PerFusion EEG software). We assessed brain activity using resting state EEG. During the EEG assessment, study participants were either seated or lying down in a quiet room for 30 minutes. If the staff observed any indications of sleep, they gently alerted the participants by stating their name. We also recorded electroopthalmogram (EOG) using electrodes placed above and below the left eye in order to monitor eye blink and movement [38]. Prior to the recording, the participant's scalp, forehead, and earlobes were prepped to ensure that impedance values were below 5 kΩ. The EEG signals were filtered using a bandpass of 0.5–70 hertz and a signal sampling rate of 500 Hz. We then multiplied the sampled epochs by a Hanning window, and the data were transformed with a Fast Fourier Transform (FFT) with Welch method. The EEG spectral analysis was performed by an EEG spectral analysis expert (SJ) who was blind to the intervention.

We computed the average power distribution for four bandwidths: delta (0–4 hertz), theta (4–8 hertz), alpha (8–13 hertz), and beta (13–30 hertz). The peak alpha band frequency (PAF) was our primary EEG outcome measurement. We calculated this as the frequency of peak alpha power (between 8 and 13 hertz) for each of 18 electrode sites (Fp1, Fp2, F7, F3, Fz, F4, F8, T3, C3, C4, T4, T5, P3, Pz, P4, T6, O1, and O2). We used MATHLAB (Mathwork, USA) for all EEG computations.

EEG was recorded on the first day of the first week (i.e., one assessment in the first baseline week), immediately after the first stimulation session and again at 24, 48, and 72 hours after stimulation (i.e., 5 EEG assessments in the first week) and then again once during the second baseline week before the second stimulation, once immediately after the second stimulation, and at 24, 48, and 72 hours after the second simulation in week 3 (i.e., a total of 10 EEG assessments).

*Peak Alpha Frequency*. Peak alpha frequency was computed using EEG data collected and processed by a trained staff. Prior to calculating PAF, the raw EEG data was processed following Coben and colleague's procedures [39]. EEG data were recorded using a 50-Hz notch filter. Thirty 2.56-s epochs were selected and artifacts were detected by EOG. Fast Fourier Transforms (FFTs) were performed on the 24 epochs (1 minute). The EOG rejection was set at 50 *µ*V. Every epoch was also visually appraised for artifacts and rejected when artifacts were deemed present.

### 3.2. Autism Treatment Evaluation Checklist (ATEC)

The ATEC scales scores were used to assess the impact of tDCS on four autism-related problem domains. The ATEC questionnaire is administered to the caregiver and yields scores in four autism problem areas: (1) speech/language/communication problems (14 items; ceiling score 28); (2) social problems (20 items; ceiling score 40); (3) sensory and cognitive awareness problems (18 items; ceiling score 36); and (4) health/physical/behavior problems (25 items; ceiling score 75). Higher scores indicate more problems within each domain. The total score can range from 0 to 179 [36]. The ATEC was administered to the participant caregivers at baseline, immediately after stimulation, and at 24-hour, 48-hour, 72-hour, and 7-day follow-ups.

### 3.3. Adverse Events

The study investigators monitored and recorded any adverse events observed during the stimulation sessions. In addition, the participants' caregivers were asked to monitor and report using open questions about any adverse events they noticed during the 24 hours following each stimulation session.

### 3.4. Statistical Analyses

For the descriptive purpose, we computed the means and standard deviations of the demographic and outcome variables. To ensure prestimulation equivalence between participants assigned to the two treatment orders (i.e., sham-active versus active-sham), we compared the PAF and ATEC scores obtained at the first baseline assessment between the two treatment order groups using paired *t*-tests. We then used repeated measures analysis of variance (ANOVA) to test the hypothesis regarding the effect of tDCS on the PAF scores with treatment order (active-sham versus sham-active), treatment condition (active versus sham), and time (baseline, immediately after stimulation, and 24-, 48-, and 72-hour follow-ups) as the independent variables. We planned to use Fisher's LSD to interpret any significant main or interaction effect found. A similar ANOVA procedure followed by LSD was used to test the study hypotheses regarding the effect of tDCS on the four ATEC scale scores as well as the total ATEC score. Finally, we computed Pearson's correlation coefficients to evaluate the association between pre- to postsessions changes in PAF assessed at the F3 electrode and changes in the ATEC scale scores as well as total ATEC score. For all analyses, *P* values of <0.05 were considered statistically significant. Analyses were completed using Stata software, version 10.0 (StataCorp, College Station, TX).

## 4. Results

A total of 24 children who were screened for possible participation between April and October 2012 met the study inclusion criteria. Four children were excluded from the study: two because of having epilepsy, one because of a skull defect, and the fourth because of a diagnosis of leukemia. Twelve right-handed and eight left-handed participants completed the entire protocol. Participants assigned to each condition order did not significantly differ with respect to age, age at diagnosis, or perinatal history. Participants continued to receive standard treatment of their autism throughout the period of the study. Specifically, five of the participants who had been taking risperidone at baseline continued to take this medication during the trial, and all of the participants received educational interventions from clinicians from different disciplines, including speech therapy, occupational therapy, and animal assisted therapy as a part of their standard care. Also, no significant differences emerged between the participants assigned to each condition order on measures of baseline PAF or on any of the ATEC scale scores. The age, handedness, age at diagnosis, perinatal history, and conventional treatment of the study participants are presented in Table 1.

### 4.1. Peak Alpha Frequency

There were no significant main or interaction effects involving condition order for PAF revealed by ANOVA analyses at any electrode. Therefore, the data were collapsed across condition order for all subsequent analyses. Repeated-measures ANOVA was used to test for the effects of active tDCS on changes in PAF over time as assessed from each of the 18 electrode sites. In these analyses, treatment condition (active versus sham tDCS) and time (baseline, immediately after treatment, and 24 hours, 48 hours, and 72 hours after treatment) were the independent variables. The results of these analyses are presented in Table 2.

**Figure 1:**
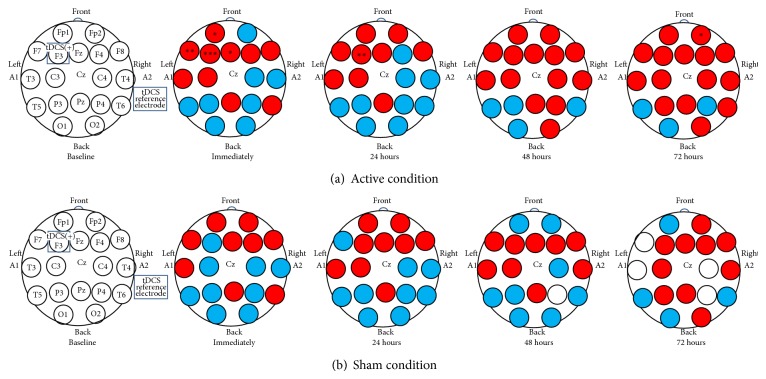
Changes in peak alpha frequency (PAF) at 18 electrode sites (referenced to Cz) after active and sham tDCS stimulation over dorsolateral prefrontal cortex (F3), relative to baseline. In the active condition, significant differences in PAF were found at 5 electrode sites: Fp1, Fp2, F7, F3, and Fc. Immediately after stimulation, PAF significantly increased at Fp1, F7, and Fc; significant increases in PAF at Fp2 were found at 72 hours after stimulation. At the stimulation site (F3), increases in PAF were found immediately and at 24 hours after stimulation. In the sham condition, PAF did not have any significant changes at any time point at any of the 18 electrode sites. Red = increase; blue = decrease; white = no changes; ∗ significant different main effect of time; ^*^ *P* < 0.05; ^**^ *P* < 0.01; ^***^ *P* < 0.001.

No significant main effects for treatment condition emerged for PAF assessed from the 17 electrode sites that were not the site of anodal stimulation (i.e., all of the sites except for F3). Significant main effects of time were found for PAF assessed from 3 of these sites: Fp2 (*F*(4,36) = 3.07; *P* = 0.019), P3 (*F*(4, 36) = 3.70; *P* = 0.007), and T6 (*F*(4,36) = 3.82; *P* = 0.006). A significant Condition × Time interaction also emerged for one of these electrode sites, Fp2 (*F*(4, 36) = 2.85; *P* = 0.026) (see Table 2). To better understand the interaction effect found, we performed post hoc analyses of the time factor for each treatment condition separately for PAF assessed from Fp2. These analyses revealed a significant baseline to 72-hour posttreatment increase in PAF (0.57, 95% CI = 0.07 to 1.06; *P* = 0.028) (Figure 1).

At the stimulation site (F3), there were significant main effects for changes in PAF for treatment condition (*F*(1, 36) = 5.28; *P* = 0.027) and time (*F*(4, 36) = 5.00; *P* < 0.001). There was also a significant Condition × Time interaction (*F*(4, 36) = 5.05; *P* = 0.001). The results of a one-way ANOVA performed to understand the time effect showed an overall increase in PAF immediately after stimulation (*F*(1,39) = 11.83; *P* = 0.001) (effect size (Cohen's *d*) = 2.33) and 24 hours (*F*(1,39) = 6.93; *P* = 0.012) (effect size = 1.75), relative to baseline, but no statistically significant increase PAF at 48 hours (*F*(1,39) = 0.52; *P* = 0.474) or 72 hours (*F*(1,39) = 0.02; *P* = 0.880) after treatment, relative to baseline (Table 2 and Figure 1).

**Figure 2:**
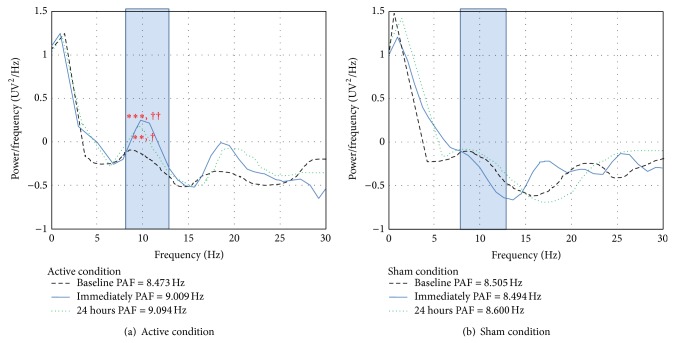
Power spectrum density at baseline relative to immediately after stimulation and 24 hours after stimulation for the two treatment conditions. (a) In the active condition, the results show significant increases in peak alpha frequency (PAF) between baseline (black dashed line) and immediately after stimulation (blue line) (*P* < 0.001). Significant increases in PAF from baseline were also found at 24 hours after stimulation (*P* = 0.004). (b) In the sham condition, no significant changes in PAF were found at either assessment point. Significant differences following active treatment, relative to baseline, are indicated by ^**^ *P* < 0.01; ^***^ *P* < 0.001. Significant differences following sham treatment, relative to baseline, are indicated by ^†^ *P* < 0.05; ^††^ *P* < 0.01.

The analyses performed to help understand the interaction effect that emerged for changes in PAF over time showed that, in the active tDCS condition, PAF increased significantly from baseline to immediately after stimulation (mean difference score = 0.54, 95% CI = 0.27 to 0.80; *P* < 0.001) and 24 hours after stimulation (mean difference score = 0.62, 95% CI = 0.23 to 1.01; *P* = 0.004). However we found no significant changes in PAF following the sham condition at any time point, relative to baseline (Figure 2).

### 4.2. Autism Treatment Evaluation Checklist (ATEC)

#### 4.2.1. ATEC Language Scale

A repeated-measures ANOVA using the ATEC language scale as the dependent variable revealed no significant main effects for condition (*F*(1,36) = 0.14; *P* = 0.711) or time (*F*(5,36) = 1.23; *P* = 0.299) and no significant Condition × Time interaction effects (*F*(5,36) = 0.57; *P* = 0.725). Consistent with these findings, post hoc analyses demonstrated no significant changes in the ATEC language scale score over time in either of the tDCS treatment conditions (Table 3(I)).

#### 4.2.2. ATEC Social Scale

The repeated-measures ANOVA performed with the ATEC social scale as the dependent variable yielded no statistically significant main effect for condition (*F*(1,36) = 1.15; *P* = 0.291). However, there was a significant main effect for time (*F*(5,36) = 2.63; *P* = 0.025) and a significant Condition × Time (*F*(5,36) = 2.69; *P* = 0.023). Analyses performed to explain the significant interaction showed that the ATEC social scale significantly decreased from baseline to 7 days after stimulation (*F*(1,39) = 6.14; *P* = 0.018) (effect size [Cohen's *d*] = 1.04) in the tDCS group. However, no significant changes over time were found for the ATEC social scale following sham tDCS treatment (see Table 3(II)). The mean difference scores for the ATEC social scale were −2.45 (95% CI = −3.38 to −1.52; *P* < 0.001) and 0.25 (95% CI = −0.61 to 1.11; *P* = 0.549) for the active and sham treatment conditions, respectively.

#### 4.2.3. ATEC Sensory and Cognitive Awareness Scale

The ANOVA analyses for the ATEC sensory and cognitive awareness scale revealed no significant main effects for condition (*F*(1,36) = 0.24; *P* = 0.626) or time (*F*(5,36) = 10.10; *P* = 0.248) and no significant Condition × Time interaction (*F*(5,36) = 0.72; *P* = 0.993). Consistent with these findings, repeated-measures ANOVAs showed no significant changes in the ATEC sensory and cognitive awareness scale over time in following either active or sham tDCS treatment (see Table 3(III)).

#### 4.2.4. ATEC Health and Behavioral Problem Scale

Analyses examining the effects of treatment condition and time on the ATEC health and behavioral problems scale revealed no significant main effect for condition (*F*(1,36) = 1.24; *P* = 0.272) but there was significant main effect for time (*F*(5,36) = 3.80, *P* = 0.003). The Condition × Time interaction was not statistically significant (*F*(5,36) = 1.28, *P* = 0.273). A one-way ANOVA revealed significantly decreases in the ATEC health and behavioral problems scale at 7 days after stimulation, relative to baseline (*F*(1,39) = 4.33; *P* = 0.044) (effect size [Cohen's *d*] = 0.66), and no significant changes in this scale following sham treatment. However, no statistically significant between-condition differences in the ATEC health and behavioral problem subscale emerged at any time point. The mean baseline to 7-day posttreatment change scores in the ATEC health and behavioral problems were −5.40 (95% CI = −8.65 to −2.15; *P* = 0.003) and −1.25 (95% CI = −3.19 to 0.69; *P* = 0.193), respectively (see Table 3(IV)).

#### 4.2.5. ATEC Total Score

The repeated-measures ANOVA with ATEC total score as the dependent variable revealed no significant main effect for condition (*F*(1,36) = 3.08; *P* = 0.088), although a significant main effect for time (*F*(5,36) = 3.78; *P* = 0.003) did emerge. The Condition × Time interaction was not statistically significant (*F*(5,36) = 1.64; *P* = 0.152).

One-way ANOVA revealed significantly changes over time in the ATEC total score for the active condition (*F*(1,39) = 11.63; *P* = 0.002) (effect size (Cohen's *d*) = 0.96); no significant changes over time emerged following sham treatment. Also, there were no statistical between-condition differences in the ATEC total score at any time point. The baseline to follow-up posttreatment changes in the ATEC total score following active tDCS stimulation were −5.05 (95% CI = −9.18 to −0.92; *P* = 0.019) at 24 hours after stimulation, −5.40 (95% CI = −10.29 to −0.51; *P* = 0.032) at 48 hours after stimulation, −6.10 (95% CI = −10.73 to −1.47; *P* = 0.013) at 72 hours after stimulation, and −7.31 (95% CI = −11.09 to −3.51; *P* = 0.001) at 7 days after stimulation. Following sham tDCS, the only significant change over time that emerged was from baseline to 48 hours after stimulation (−3.25, 95% CI = −6.48 to −0.02; *P* = 0.049; see Table 3(V)).

We also found between-group differences in the ATEC total score immediately after treatment −4.00 (95% CI = −1.13 to −6.87; *P* = 0.009), 24 hours after stimulation −4.85 (95% CI = −2.00 to −7.70; *P* = 0.002), 48 hours after stimulation −3.85 (95% CI = −0.93 to −6.77; *P* = 0.013), 72 hours after stimulation −5.20 (95% CI = −2.20 to −8.20; *P* = 0.002), and 7 days after stimulation −8.45 (95% CI = −5.92 to −10.98; *P* < 0.000).

### 4.3. Correlation between Changes in PAF and Changes in ATEC Scale Scores

**Figure 3:**
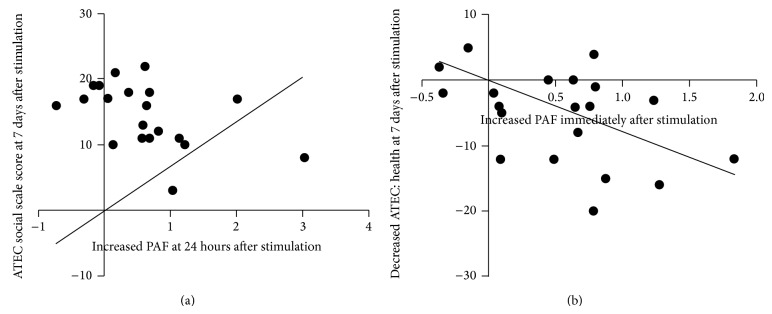
Correlation between change in peak alpha frequency (PAF) and autism treatment evaluation checklist (ATEC) subscale scores. (a) An increase in PAF at 24 hours after stimulation and change in the ATEC social scale at 7 days after stimulation (*r* = −0.47, *P* = 0.037). (b) An increase in PAF immediately after stimulation and change in the ATEC health and behavioral problem scale at 7 days after stimulation (*r* = −0.46, *P* = 0.039).

The correlations between changes in PAF measured from F3 and changes in the ATEC scales from baseline to each time point indicated significant associations for the total ATEC score, the ATEC social score, and the ATEC health and behavioral problem score following active tDCS treatment. Specifically, we found that increases in PAF assessed at F3 from baseline to immediately after stimulation were negatively associated with baseline to 7-day decreases in the ATEC health and behavioral problems scale (*r* = −0.46, *P* = 0.039), and baseline to 24-hour increases in PAF were negatively associated with baseline to 7-day decreases in the ATEC social scale score (*r* = −0.47, *P* = 0.037; see Figure 3).

### 4.4. Adverse Events

No serious adverse events were found in any of the participants. The only minor adverse event that was observed was transient erythematous rash, which occurred in three of the participants who received active tDCS and which always cleared within 10 minutes after stimulation.

## 5. Discussion

This study revealed that a single stimulation of anodal tDCS over the left DLPFC (F3 in the international 10/20 system) resulted in significantly greater increases in PAF measured from the F3 electrode (as well as from three nearby electrodes) that is maintained for 24 hours among participants in the active tDCS condition, relative to those in the sham tDCS condition. Also as hypothesized, we found a significant association between improvements in the ATEC social and health and behavioral problems subscale and an increase in PAF in those who received active tDCS treatment.

The findings demonstrating an increase in PAF from pre- to immediately postanodal tDCS stimulation at and near the site of stimulation (F3) provide preliminary evidence for a possible mechanism of the effects of tDCS on a measure previously found to be associated with autistic severity in individuals with ASD. This finding is also consistent with those indicating that (1) anodal tDCS over F3 improves language acquisition after the stimulation session in individuals with ASD, relative to baseline [33], and (2) significant improvements in measures of autism severity and symptoms occur at 7 days after treatment after 1 mA of anodal tDCS applied over F3 for 20 minutes on 5 consecutive days [34].

Because this is the first study that we are aware of examining the effects of tDCS on EEG spectral measures in individuals with ASD, a comparison with previous results regarding these effects is not possible. With regard to the pathophysiology of ASD, one previous study has shown reduced alpha coherence between the frontal cortex and the temporal, parietal, and occipital cortices in individuals with ASD, relative to controls [24]. Another has found weaker left frontal-temporal connectivity in a sample of individuals with ASD, relative to controls [40].

These findings are consistent with results from multiple fMRI studies that have shown a reduction in resting state frontotemporal connectivity in individuals with ASD [18, 41, 42]. Similarly, a positron emission tomography study of resting state brain activity in a sample of adult with ASD has shown reduced correlations in glucose metabolism between frontal and other cortical areas [43]. Another study of resting state brain activity in individuals with ASD using fMRI has shown generalized decreases in frontoparietal and frontooccipital connectivity [19, 44]. As a group, these studies suggest that the connectivity between the frontal lobe and other cortical regions may be weakened in individuals with ASD. The frontal lobe is known to play an important role in higher-order cognitive, social, and emotional functioning [30]. It should therefore not be surprising that deficits in frontal lobe connectivity are reported in individuals with ASD [45–47].

Our findings indicate that increasing cortical activity in left frontal regions (as reflected by an increase in PAF) may be associated with improvements in some ASD problem areas, specifically social problems and health and behavior problems as assessed by the ATEC. This finding, if replicated, provides further support for the idea of disruptions in the activity of frontal brain regions may underlie some ASD symptoms and the possibility that brain stimulation techniques as a potential treatment for ASD. There are a variety of brain stimulation techniques that could be explored. For example, high frequency rTMS has been shown to result in increases in PAF at F3, F4, C3, T3, T4, Fz, and Cz. Similar to our findings using tDCS, rTMS stimulation has the largest effects at the stimulation site as well as surrounding sites, with weaker effects as the distance from the stimulation site grows [48]. As a group, this evidence indicates that the effects of brain stimulation techniques are specific to the site of stimulation and so could potentially be used to target activity in specific regions of interest.

There are a number of limitations that should be considered when interpreting the results of this study. First, resting EEG can be influenced by a large number of factors, making the identification of specific patterns in groups of patients with ASD challenging. However, we still found significant effects despite this challenge, perhaps related to the use of a crossover design which could reduce the effects of some confounding factors. In addition, although we used standard procedures (i.e., the international 10-20 system) for electrode placement, we did not confirm that the electrode was directly over the DLPFC, which was our primary area of interest. Moreover, given the size of the electrodes (35 cm^2^), tDCS procedures likely result in more generalized (hemicortical) stimulation than very specific stimulation. Thus, we cannot confirm that the DLPFC—and only the DLPC—was stimulated in this study. It therefore remains possible that changes in activity in areas other than the DLPFC may explain or underlie the benefits found. In addition, we did not image brain activity before and after treatment, so we are not able to examine or confirm any findings related to possible neurophysiological mechanisms of tDCS's benefits. Another issue to consider in understanding the effects of tDCS involves the potential interaction of tDCS with medications. Very little is known about how the presence or absence of different medications might influence the effects of tDCS treatment and how tDCS treatment might influence (i.e., enhance or attenuate) the impact of medications. Unfortunately, given the relatively low number of subjects who were taking any one type of medication in our sample, it was not possible to examine these potential moderation effects in the current study. This would be an important focus of future research. Finally, given the small sample size, as well as the use of multiple statistical tests, there is an increased risk in this study for type I errors (i.e., identifying a significant effect in the sample when such an effect does not exist in the population). Replication of the current findings, ideally using larger samples of subjects if possible, is needed to determine their reliability and generalizability. Despite these limitations, to our knowledge this is the first study to demonstrate that anodal tDCS over F3 may have immediate effects on PAF and may improve functioning in important ASD symptoms and problems areas. Anodal tDCS may be an alternative to medication management for some families or patients who are not interested in or decline psychopharmacological treatment. In addition, nowadays, there is still no specific medication for treatment of the causes of autism. Anodal tDCS over F3 may be one of the specific therapies. Further research is needed to examine the potential of brain stimulation strategies as a treatment for ASD and to help clarify the mechanisms of these treatments using neuroimaging techniques.

## Figures and Tables

**Table 1:** Descriptive data for the study participants (*n* = 20).

ID	Sex	Age (years)	Handedness	Age of diagnosis (months)	Parturition	Conventional treatment	
						Medication	Behavioral therapy
1	Male	6	left	24	C-section	PN, MP, RD	DS, ST
2	Male	8	right	30	C-section	—	OT
3	Male	5	right	36	Natural	—	DS, OT
4	Male	5	right	31	C-section	—	DS, OT
5	Male	7	right	24	C-section	RD	DS, OT
6	Male	5	left	32	C-section	—	DS, OT, AT (horse)
7	Male	6	left	36	Natural	—	DS, OT, ST
8	Male	6	left	34	Natural	—	DS, OT, ST
9	Male	8	right	26	C-section	—	DS, OT, ST
10	Male	5	right	24	Natural	RD	DS, ST
11	Male	7	right	18	Natural	—	DS, ST
12	Male	6	right	35	Natural	—	DS, OT, ST
13	Male	6	left	35	C-section	—	DS, ST
14	Male	6	left	29	Natural	RD	DS, ST
15	Male	8	right	38	C-section	—	DS, ST
16	Male	7	right	20	C-section	—	DS, OT, ST
17	Male	8	left	40	Natural	—	DS, ST
18	Male	7	right	36	C-section	RD	DS, OT, ST
19	Male	7	right	32	C-section	—	DS, OT, ST
20	Male	5	left	28	C-section	—	DS, ST

DS: developmental stimulation, ST: speech therapy, AT: animal assisted therapy, OT: occupational therapy, PN: pyritinol, MP: methylphenidate, and RD: risperidone.

**Table 2:** Peak alpha frequency (PAF) scores of EEG recorded from different electrode sites.

Channel	Active condition					Sham condition					Remark
	Baseline	Immediately	24 hours	48 hours	72 hours	Baseline	Immediately	24 hours	48 hours	72 hours	
Fp1	8.98	9.34^*^	9.05	8.84	9.17	8.96	8.98	8.99	8.95	8.95	
Fp2	8.93	8.80	9.02	9.03	9.50^*^	8.96	8.98	8.98	8.95	8.98	†
F7	8.88	9.39^**^	9.19	9.08	9.19	8.85	8.86	8.83	8.87	8.85	
F3	8.47	9.01^***^	9.09^**^	8.69	8.54	8.51	8.49	8.60	8.58	8.55	††
Fz	8.94	9.34^*^	9.18	9.04	9.23	8.88	8.89	8.89	8.89	8.91	
F4	8.83	9.09	8.81	9.00	9.08	8.82	8.84	8.83	8.83	8.84	
F8	8.77	9.01	9.02	8.84	9.15	8.76	8.78	8.82	8.77	8.79	
T3	8.83	9.08	8.91	8.97	9.09	8.82	8.86	8.83	8.83	8.82	
C3	8.98	9.17	9.07	9.11	9.21	8.87	8.84	8.93	8.97	8.90	
C4	9.39	9.29	9.24	9.44	9.42	9.35	9.27	9.28	9.32	9.35	
T4	9.01	8.98	8.98	9.18	9.24	8.99	8.97	8.95	9.00	9.09	
T5	9.49	9.00	9.16	9.17	9.36	9.40	9.36	9.35	9.24	9.35	
P3	9.49	9.14	9.21	9.27	9.60	9.40	9.38	9.34	9.38	9.43	
Pz	9.12	9.25	9.25	9.19	9.36	9.12	9.14	9.13	9.14	9.23	
P4	9.43	9.19	9.20	9.55	9.33	9.30	9.24	9.20	9.30	9.30	
T6	9.23	9.48	9.07	9.08	9.24	9.17	9.22	9.09	9.10	9.12	
O1	9.70	9.39	9.41	9.54	9.54	9.65	9.62	9.57	9.59	9.55	
O2	9.53	9.40	9.26	9.70	9.59	9.50	9.47	9.45	9.49	9.52	

Significant difference when compared with baseline indicated by ^*^ *P* < 0.05; ^**^ *P* < 0.01; ^***^ *P* < 0.001.

Significant Condition × Time interactions when compared between active and sham condition indicated by ^†^ *P* < 0.05; ^††^ *P* < 0.01.

**Table 3:** Means and standard deviations of the autism treatment evaluation checklist (ATEC) scale scores at each assessment point for active and sham conditions.

Autism treatment evaluation checklist	Active	Sham	*P* value
(I) Language scale			
Baseline	10.80 ± 5.49	10.90 ± 4.61	0.951
Immediately after treatment	10.05 ± 4.36	10.85 ± 4.28	0.562
24 hours after treatment	9.80 ± 4.23	10.95 ± 4.52	0.411
48 hours after treatment	10.25 ± 5.09	10.55 ± 4.20	0.840
72 hours after treatment	9.85 ± 4.73	10.35 ± 4.21	0.726
7 days after treatment	10.75 ± 5.15	10.95 ± 5.03	0.902
(II) Social scale			
Baseline	17.00 ± 4.41	17.55 ± 2.76	0.639
Immediately after treatment	16.90 ± 3.49	17.40 ± 3.63	0.660
24 hours after treatment	15.85 ± 4.28	16.60 ± 4.41	0.588
48 hours after treatment	15.70 ± 3.77	16.55 ± 3.58	0.469
72 hours after treatment	15.60 ± 5.34	17.10 ± 3.39	0.296
7 days after treatment	14.55 ± 4.98	17.80 ± 3.11	0.018^†^
(III) Sensory and cognitive awareness scale			
Baseline	20.50 ± 4.22	21.50 ± 3.40	0.414
Immediately after treatment	20.10 ± 4.60	20.60 ± 4.85	0.740
24 hours after treatment	20.20 ± 4.36	20.55 ± 4.57	0.806
48 hours after treatment	20.00 ± 5.40	20.30 ± 4.64	0.851
72 hours after treatment	20.45 ± 4.02	21.05 ± 4.29	0.650
7 days after treatment	21.10 ± 4.89	21.90 ± 4.44	0.591
(IV) Health and behavioral problems scale			
Baseline	20.70 ± 8.25	20.75 ± 7.22	0.984
Immediately after treatment	18.50 ± 7.34	20.70 ± 6.59	0.325
24 hours after treatment	18.10 ± 7.91	20.70 ± 6.48	0.263
48 hours after treatment	17.65 ± 9.00	20.05 ± 8.30	0.386
72 hours after treatment	17.00 ± 7.28	19.60 ± 6.37	0.237
7 days after treatment	15.30 ± 6.45	19.50 ± 6.32	0.044^†^
(V) Total ATEC score			
Baseline	69.00 ± 10.48	70.70 ± 9.22	0.589
Immediately after treatment	65.55 ± 9.94	69.55 ± 10.41	0.222
24 hours after treatment	63.95 ± 10.53	68.80 ± 10.35	0.150
48 hours after treatment	63.60 ± 10.96	67.45 ± 9.95	0.252
72 hours after treatment	62.90 ± 8.65	68.10 ± 7.91	0.054
7 days after treatment	61.70 ± 6.70	70.15 ± 8.83	0.002^††^

Data are presented as mean ± S.D.

Significant differences between the two conditions indicated by ^†^ *P* < 0.05; ^††^ *P* < 0.01.
